# Wide distribution of *Trypanosoma cruzi*-infected triatomines in the State of Bahia, Brazil

**DOI:** 10.1186/s13071-019-3849-1

**Published:** 2019-12-26

**Authors:** Gilmar Ribeiro, Carlos G. S. dos Santos, Fernanda Lanza, Jamylle Reis, Fernanda Vaccarezza, Camila Diniz, Diego Lopes Paim Miranda, Renato Freitas de Araújo, Gabriel Muricy Cunha, Cristiane Medeiros Moraes de Carvalho, Eduardo Oyama Lins Fonseca, Roberto Fonseca dos Santos, Orlando Marcos Farias de Sousa, Renato Barbosa Reis, Wildo Navegantes de Araújo, Rodrigo Gurgel-Gonçalves, Mitermayer G. dos Reis

**Affiliations:** 1 Instituto Gonçalo Moniz, Fiocruz-BA, Laboratório de Patologia e Biologia Molecular, Rua Waldemar Falcão, 121, Candeal-Salvador, BA CEP: 40296–710 Brazil; 2 SESAB/Diretoria de Vigilância Epidemiológica (DIVEP), Centro de Atenção à Saúde José Maria de Magalhães Netto., Av. Antônio Carlos Magalhães, s/nº, Parque Bela Vista, Salvador, BA CEP 41.820–000 Brazil; 3 SESAB/Laboratório Central de Saúde Pública Prof, Gonçalo Moniz LACEN, Rua Waldemar Falcão, 12, Candeal, Salvador, BA 40296–710 Brazil; 4 Instituto de Tecnologias da Saúde (CIMATEC ITS), Av. Orlando Gomes, 1845 Piatã, Salvador, BA 41650–010 Brazil; 5 Faculdade de Medicina-UFBA, Rua Reitor Miguel Calmon, s/n Vale do Canela, Salvador, BA 40110–100 Brazil; 6 Laboratório de Parasitologia Médica e Biologia de Vetores Faculdade de Medicina, Universidade de Brasília, Campus, Universitário Darcy Ribeiro, Asa Norte, Brasília, Distrito Federal 70910–900 Brazil; 7 Núcleo de Medicina Tropical, Faculdade de Medicina, Universidade de Brasília, Campus Universitário Darcy Ribeiro, s/n, Asa Norte, Brasília, Distrito Federa 70910–900 Brazil; 8 Coordenação-Geral de Vigilância de Zoonoses e Doenças de Transmissão Vetorial, Secretaria de Vigilância em Saúde, Ministério da Saúde, SRTV 702, Via W 5 Norte, Brasília, DF 70723–040 Brazil; 9 Faculdades Ruy Barbosa-Wyden, Rua Theodomiro Baptista, 422 - Rio Vermelho, Salvador, BA 41940–320 Brazil; 10 Universidade Salvador-UNIFACS, Salvador, Brazil; 11 Yale University, New Haven, CT 06520 USA

**Keywords:** Chagas disease, Entomological surveillance, *Trypanosoma cruzi*, Blood meal, PCR

## Abstract

**Background:**

The identification of *Trypanosoma cruzi* and blood-meal sources in synanthropic triatomines is important to assess the potential risk of Chagas disease transmission. We identified *T. cruzi* infection and blood-meal sources of triatomines caught in and around houses in the state of Bahia, northeastern Brazil, and mapped the occurrence of infected triatomines that fed on humans and domestic animals.

**Methods:**

Triatominae bugs were manually captured by trained agents from the Epidemiologic Surveillance team of Bahia State Health Service between 2013 and 2014. We applied conventional PCR to detect *T. cruzi* and blood-meal sources (dog, cat, human and bird) in a randomized sample of triatomines. We mapped triatomine distribution and analyzed vector hotspots with kernel density spatial analysis.

**Results:**

In total, 5906 triatomines comprising 15 species were collected from 127 out of 417 municipalities in Bahia. The molecular analyses of 695 triatomines revealed a ~10% *T. cruzi* infection rate, which was highest in the *T. brasiliensis* species complex. Most bugs were found to have fed on birds (74.2%), and other blood-meal sources included dogs (6%), cats (0.6%) and humans (1%). *Trypanosoma cruzi*-infected triatomines that fed on humans were detected inside houses. Spatial analysis showed a wide distribution of *T. cruzi*-infected triatomines throughout Bahia; triatomines that fed on dogs, humans, and cats were observed mainly in the northeast region.

**Conclusions:**

Synanthropic triatomines have a wide distribution and maintain the potential risk of *T. cruzi* transmission to humans and domestic animals in Bahia. Ten species were recorded inside houses, mainly *Triatoma sordida*, *T. pseudomaculata,* and the *T. brasiliensis* species complex. Molecular and spatial analysis are useful to reveal *T. cruzi* infection and blood-meal sources in synanthropic triatomines, identifying areas with ongoing threat for parasite transmission and improving entomological surveillance strategies.

## Background

Chagas disease is the most frequent cause of heart failure in rural populations in vector-endemic countries in Latin America [1, 2]. It is an infection caused by *Trypanosoma cruzi* (Chagas, 1909), a protozoan transmitted by blood-feeding bugs [3]. No vaccines or effective antiparasitic treatments are available to cure Chagas cardiomyopathy, so vector surveillance and control are the main strategies to prevent human infection in areas with vectorial transmission [4].

In Brazil, the control of Chagas disease vectors was implemented systematically between 1975 and 1983 when the main vector, *Triatoma infestans* (Klug, 1834), infested domiciles in 12 states. In 1991, Brazil integrated an international consortium to reduce vectorial transmission through insecticide spraying [4, 5]. The systematic actions of chemical treatment were effective; in 2006, the World Health Organization (WHO) certified Brazil as free of *T. cruzi* transmission by *T. infestans*. However, some recent outbreaks have been associated with the oral transmission, mainly due to açai palm juice consumption and other *T. cruzi-*contaminated food in the Brazilian Amazon, where *Rhodnius* species are frequent [6]. Moreover, new cases of vector-borne Chagas disease transmitted by either sylvatic vectors invading houses or domestic/peridomestic populations [e.g. *T. brasiliensis*, *T. pseudomaculata*, *T. sordida*, *Panstrongylus megistus* (Burmeister, 1835)] are being recorded in Brazil [7, 8]. Epidemiological data show 2.2 deaths per 100,000 inhabitants in 2017 in Brazil and the highest value was recorded at Goiás State (22.4 deaths per 100,000 inhabitants). Between 2007 and 2016, 35 Brazilian municipalities accounted for 85% of confirmed cases in the Notification Disease Information System (SINAN). Of these 35 municipalities, 99.5% are located in the Amazon region and 87% in the State of Pará (Additional file 1: Figure S1). Most of the new confirmed acute Chagas disease cases notified to the Brazilian Ministry of Health were classified as oral transmission [8]. The presence of ~60 species of native vectors in Brazil [9] in a wide endemic area of Chagas disease with different transmission scenarios [10] and a progressive reduction of the human and financial resources needed to sustain the continuity of the control actions, highlight the need for updated studies about surveillance of triatomines in Brazilian states.

Endemic areas for *T. cruzi* transmission in the state of Bahia were described a few years after the discovery of Chagas disease and were mainly associated with *P. megistus* [11] and *T. infestans* [12]. More recently, outbreaks of *T. cruzi* transmission associated with *T. sordida* showed the potential role of this species to transmit *T. cruzi* to humans [13, 14]. In addition, residual foci of *T. infestans* were found in Bahia [15] and one acute case of Chagas disease was confirmed in 2018 (Additional file 1: Table S1).

In Bahia, 26 triatomine species have been registered [9, 16]; most are strictly associated with the wild environment or peridomiciles, but others are detected inside houses where they feed on domestic animals and humans [15, 17–21]. The identification of *T. cruzi* infection and blood-meal sources in synanthropic triatomines is important to assess the potential risk of Chagas disease transmission in human dwellings. Here, we identified *T. cruzi* infection and blood-meal sources of triatomines caught at different environments in Bahia, northeast Brazil, and mapped the occurrence of infected triatomines that fed on humans and domestic animals between 2013 and 2014.

## Methods

### Study area

**Fig. 1:**
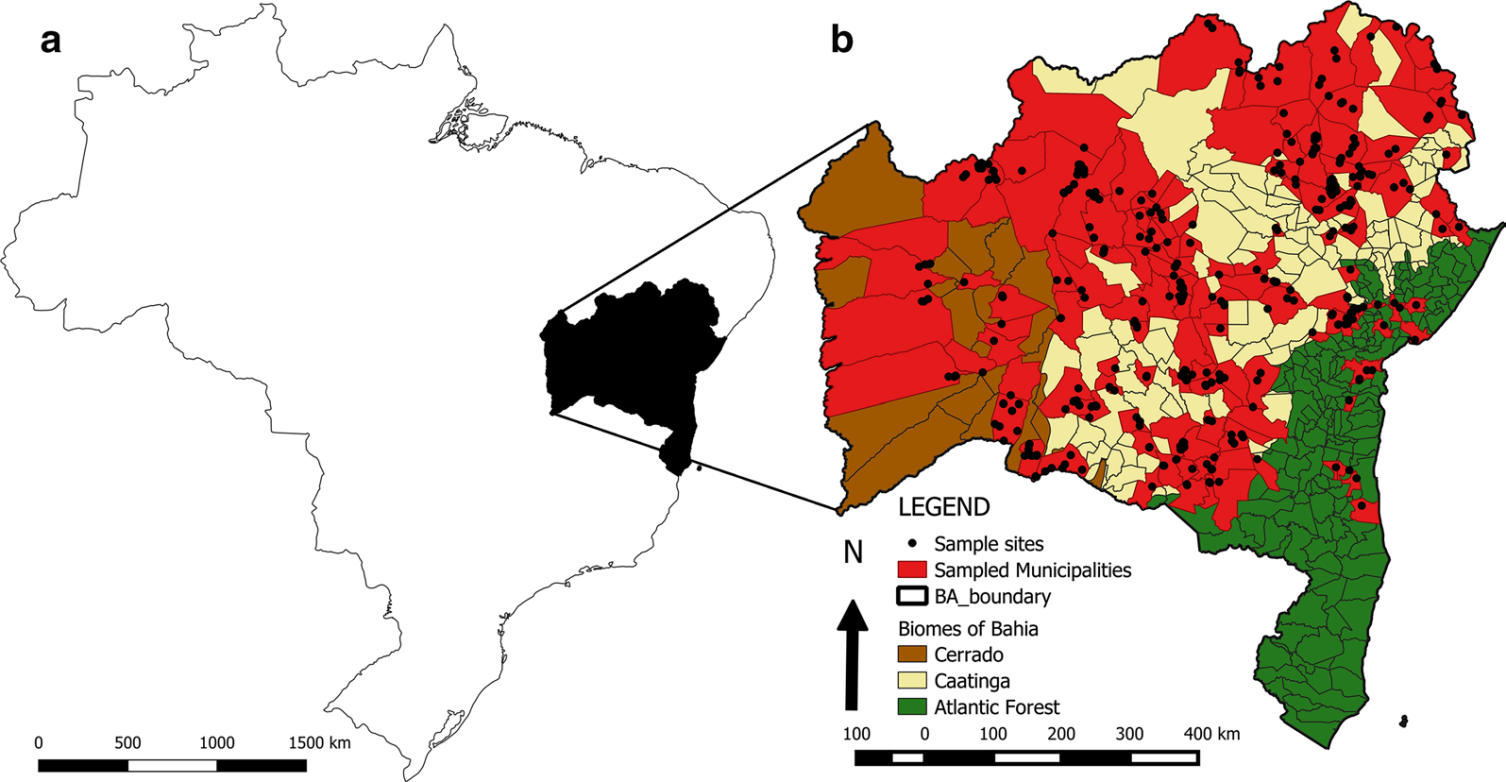
Study area. **a** Geolocation of Bahia State in Brazil. **b** Sampled municipalities (polygons) and sample locations (black dots) of triatomines collected between 2013–2014

The state of Bahia has 417 municipalities, and it is situated in the northeast region of Brazil (Fig. 1). In the western region of Bahia, the Cerrado is the main biome, with relatively high precipitation between 300 and 800 mm and a tropical climate. A tropical climate of altitude is present in the region of Chapada Diamantina; however, in the semiarid region, where the Caatinga biome predominates, rainfall indices are very low, between 100 and 300 mm, and there are long dry periods. On the marine coast, annual rainfall can exceed 1500 mm, and the main biome is Atlantic Forest.

### Insect collection

**Fig. 2:**
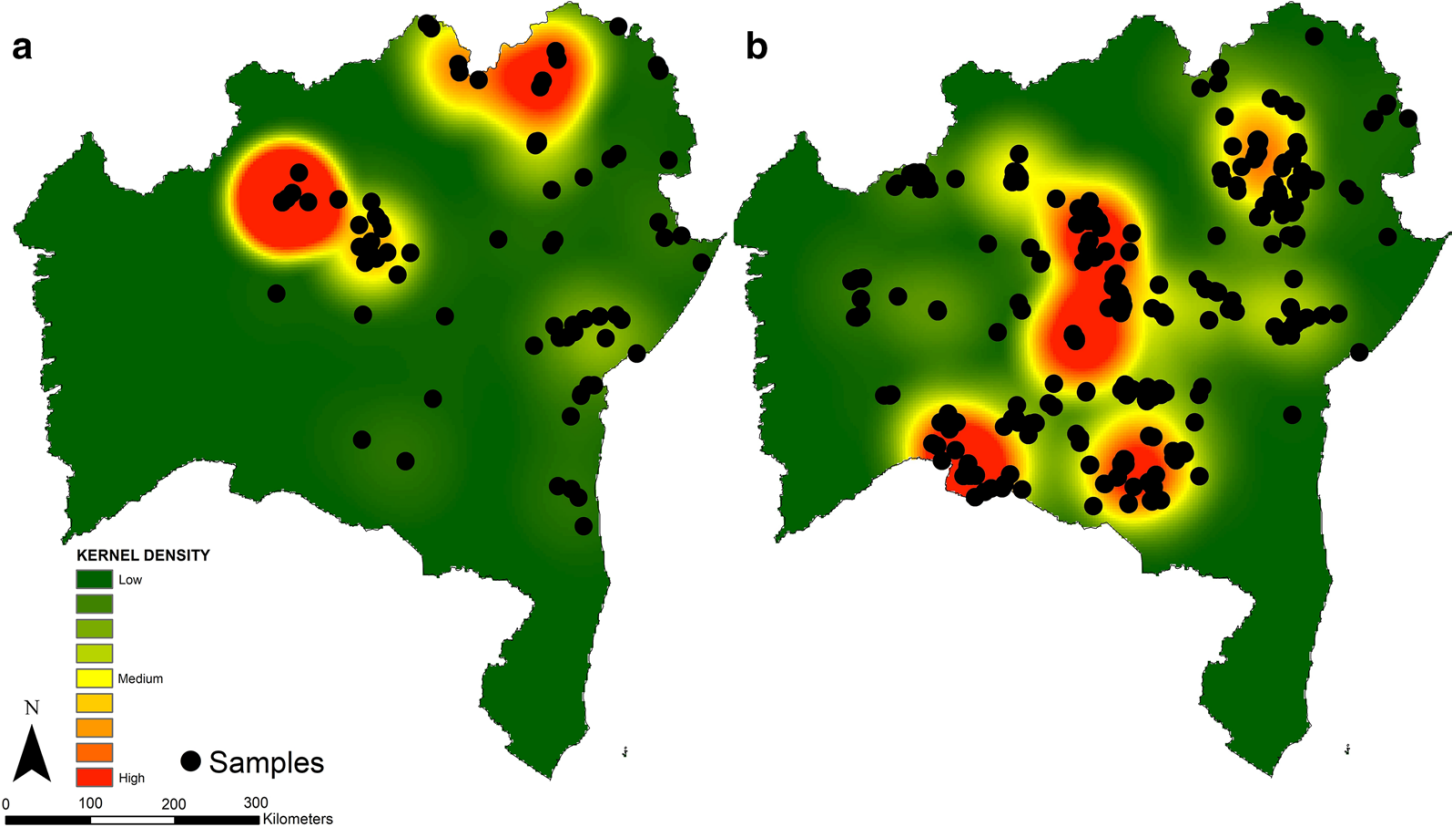
Maps showing the location of triatomine sampling points in the State of Bahia, Brazil, between 2013–2014. **a** Intradomestic environment. **b** Peridomestic environment. The heat gradient represents areas with the highest density of vectors by kernel density with a radius of 2.4 km

Geographical information system (GIS) data and triatomine bugs were obtained by trained agents from the Epidemiologic Surveillance team of Bahia State Health Service (SESAB) and IGM/FIOCRUZ-BA. Triatomines were captured monthly between 2013–2014. The collections were carried out as established by the National Programme for the Control of Chagas disease [22] in the localities with a prior history of infestation by triatomines (Fig. 2) and were part of the regular activity of the technicians of the health programs of Bahia health surveillance system. The inspections of the internal and external walls of the residence and inside rooms of the house unit and its annexes were carried out following the standardized inspection protocol of Brazil ministry of health [23].

Health agents performed exhaustive sampling and captured all the specimens found inside houses and at the peridomestic environment (kennel, cattery, corral, etc.). Additionally, triatomines were collected from the wild environment (away from human settlements) to serve as negative controls in the analyses of blood-meal sources, as DNA of humans and domestic animals (cats, dogs, and humans) was not expected in wild bugs. Metal forceps and flashlights were used to survey crevices and nonluminous sites. We selected the sampled municipalities according to epidemiological priority criteria and the capacity of SESAB at each municipality to carry out the program activities. The teams carried out collections based on the surveillance strategy of the Chagas Disease Control Programme of SESAB.

Triatomines were identified using specific identification keys [24]. Then, we photographed dorsally and ventrally, and dissected the bugs, separating head and wings, legs, and abdomen. Samples were stored in 70% alcohol + 5% glycerin at 5 °C and labeled with a QR code. We carried out a blind identification process at LACEN-BA and FIOCRUZ-BA, independently, and the teams discussed conflicting identifications individually. In addition, voucher specimens of triatomines were deposited into the SESAB entomological collection, as a reference of triatomine vectors, and all images taken of the insects are available for consultation.

### Molecular procedures

Triatomines that were dead/dry or stored incorrectly and first- and second-stage nymphs were not included in the molecular analyses to increase the efficacy of DNA extraction with a DNAzol commercial kit (DNAzol; Gibco BRL/Life Technologies, Gaithersburg, MD, EUA). We dissected triatomines in a biological safety cabinet to avoid contamination of the sample with human DNA. We macerated abdomen samples with TissueLyser L-Beader (Loccus, São Paulo, Brazil), plastic disposable maceration pistils and metal beads. Each sample was kept in a 2 ml autoclaved conical tube with a screw cap, and 1 ml of DNAzol and five autoclaved stainless-steel metal beads were added following the DNAzol standard protocol.

After purification, we quantified the DNA with a NanoDrop™ Spectrophotometer and the samples were set to a concentration of ~100 ng/µl. Conventional [25] and multiplex [26, 27] PCRs were performed with specific primers to detect *T. cruzi* based on mini-exon genes and the cytochrome *c* oxidase subunit 2 (*cox*2) gene.

To confirm that good-quality DNA was present in the samples, we amplified a 127-bp fragment of the ITS2 nuclear rDNA marker [28]. For the amplification of molecular targets, a commercial kit with Qiagen PCR Master Mix (QIAamp, Qiagen, Hilden, Germany) was used in a Mastercycler Gradient thermocycler (Eppendorf, Foster City, California, USA). The PCR conditions and primers are described in Additional file 1: Tables S2–S4.

Samples of *T. cruzi* cultures were obtained from the Experimental Chagas Disease Laboratory (LACEI/CPqGM) and used for positive controls. The DNA samples of dogs (*Canis lupus familiaris L.*), birds (*Gallus gallus L.*) and cats (*Felis catus L.*) were obtained from the blood of healthy animals from the laboratory. The human blood sample was obtained from researchers of the team (GR and CGSS). All the samples used as controls had the DNA purity evaluated with a Nanodrop^TM^ spectrophotometer and adjusted to a concentration of ~100 ng/µl. Then, the samples were aliquoted and kept at − 70 °C until use.

During the standardization of the PCR, amplified products of the PCR (10 μl) were separated by electrophoresis in an agarose gel, stained with SYBR Safe (Invitrogen, CA, USA), visualized with blue light and photographed with a Photo-documenter MultiDoc-it (UVP, Imaging Systems, Upland, CA, USA). Images were analyzed with UVP GelStudio™ (VisionWorks, CA, USA) software. Before standardization, we analyzed PCR results by capillary electrophoresis in an Applied Biosystems ABI-3500 DNA sequencer [29]. The generated electropherograms were analyzed with GeneScan analysis software version 3.1.

### Statistical analysis and mapping procedures

*Trypanosoma cruzi* infection and blood-meal frequencies were compared between triatomine species and habitats (intra-, peridomestic, wild environment) by the Chi-square or Fisherʼs exact tests using the StartCalc tool in EpiInfo™. For statistical analyses, 95% confidence intervals (CI) and *P*-values (< 0.05) were evaluated. Records of triatomines in Bahia were referenced to geographical coordinates using a GPS. When there was no information on the specific GIS coordinates, we calculated the municipality centroid using ArcGIS/ArcMap 10.5 software which was also used to map triatomine spatial distribution.

We analyzed vector hotspots with kernel density spatial analysis. In order to determine if the spatial pattern of the data is either clustered, dispersed or random, the spatial autocorrelation was evaluated by global Moranʼs index (I), *z*-score and *P*-value statistics interpretation in the Spatial Autocorrelation tool [58]. To determine the appropriate distance threshold or radius to elaborate kernel density analysis, we used the Incremental Spatial Autocorrelation tool [58]. We used the vector measures spatial autocorrelation for a series of distance increments and reports, for each distance increment, the associated Moranʼs index, expected index, variance, *z*-score, and *P*-value. Peaks in *z*-scores reflect distances where the spatial processes promoting clustering are most pronounced. Hotspots were represented by the Kernel Density tool [58]. The layers (.shp) used during this study were obtained from the IBGE website (https://downloads.ibge.gov.br/).

## Results

In total, we collected 5906 triatomines belonging to 15 species from 127 of 417 municipalities in Bahia. Most of them (*n* = 4640) were collected in 823 household units (intra- and peridomestic environments), especially in peridomestic areas (90%). The distributions of sampled triatomines by species, collection environments and municipalities are shown in Table 1.

**Table 1:** Triatomines (*Panstrongylus* spp., *Psammolestes* spp. and *Triatoma* spp.) collected in different environments in Bahia State, Brazil, between 2013–2014

Species	Intradomestic			Peridomestic			Wild environment			Not determined			Total	Distribution		
	AM	AF	N	AM	AF	N	AM	AF	N	AM	AF	N		*n*	%	H
*P. geniculatus*	3	8	0	3	0	0	0	0	0	0	0	0	14	9	7.32	10
*P. lutzi*	2	4	0	4	4	0	0	0	0	1	1	0	16 (3)	4	3.25	10
*P. megistus*	0	3	0	6	7	2	0	0	0	3	2	0	23 (6)	5	4.07	7
*Ps. tertius*	0	0	0	6	8	0	0	0	0	0	0	0	14 (8)	1	1.63	1
*T. brasiliensis*	23	16	0	19	24	44	10	14	27	1	0	0	178 (57)	20	16.26	41
*T. juazeirensis*	32	20	89	12	17	13	1	2	0	8	13	20	227 (96)	7	5.69	4
*T. melanica*	0	0	0	4	3	12	0	0	0	0	0	0	19 (7)	1	0.81	1
*T. infestans*	0	0	0	45	102	294	0	0	0	0	0	0	441 (80)	1	0.81	1
*T. lenti*	0	0	0	0	0	1	1	0	5	0	0	0	7 (2)	1	0.81	12
*T. melanocephala*	6	6	0	1	2	0	0	0	0	1	1	0	17 (1)	12	9.76	12
*T. petrocchiae*	0	1	0	0	0	0	0	0	0	0	0	0	1 (1)	1	0.81	1
*T. pseudomaculata*	23	16	25	117	166	250	0	0	0	33	40	244	914 (81)	45	36.59	89
*T. sordida*	89	103	122	712	773	1370	0	0	0	51	60	296	3576 (293)	70	56.91	267
*T. sherlocki*	0	0	0	0	0	0	70	199	154	0	0	0	423 (58)	1	0.70	^a^
*T. tibiamaculata*	9	10	0	3	6	0	0	0	0	2	6	0	36 (3)	7	5.69	26
Total	187	187	236	932	1112	1986	82	215	186	100	123	560	5906 (696)	127	100	482

^a^From sylvatic environment

*Abbreviations*: *n*, number of municipalities with triatomine occurrence; AM, adult male; AF, adult female; N, Nymph; (), selected samples for molecular biology experiments; H, number of houses with triatomine occurrence

We collected 610 specimens of 10 species inside domiciles in 55 municipalities. We detected colonies of *T. sordida*, *T. pseudomaculata* and *T. juazeirensis* in houses mainly in municipalities in the Caatinga biome. We collected 4030 specimens of 13 species in peridomiciles of 97 municipalities. *Triatoma sordida* was the most captured and widely distributed species in Bahia State, followed by *T. pseudomaculata* (Table 1). We detected a colony of *T. infestans* with more than 400 specimens inside a chicken coop, five meters from a household. We also captured 484 triatomines of four species in the wild environment in four municipalities.

**Fig. 3:**
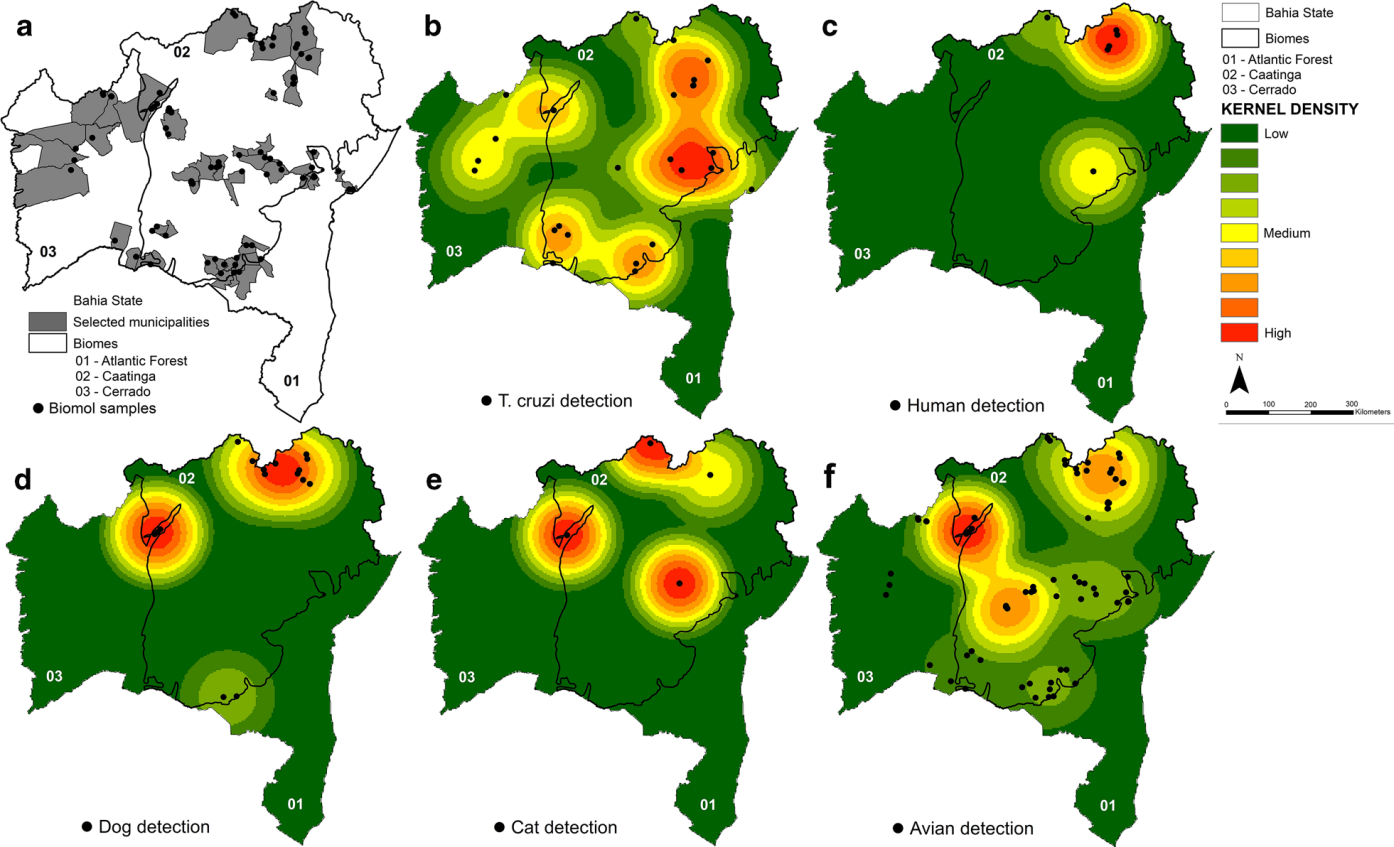
Maps showing the distribution of triatomines by *Trypanosoma cruzi* infection and blood-meal sources in the State of Bahia, Brazil, between 2013–2014. The grey lines and numbers show the limits of the biomes in Bahia State. **a** Distribution of all triatomines used in molecular detection. **b** *T. cruzi-*infected triatomines. **c**–**f** Spatial distribution of triatomines fed on humans, dogs, cats, and birds. The heat gradient represents areas with the highest density of vectors by kernel density with a radius of 2.4 km

We selected 696 triatomines for molecular evaluation of *T. cruzi* infection and blood-meal analysis (Table 1). A total of 99.85% (*n* = 695) showed specific amplification for triatomine DNA with an ITS2 nuclear rDNA marker, indicating the DNA integrity of the samples. All molecular targets evaluated have shown a spatial pattern expressed as clustered and showed an appropriate radius of 1.86 km (*T. cruzi*), 2.11 km (human), 1.98 km (dog), 1.77 km (cat), 1.98 km (bird) (Fig. 3). Almost 10% (*n* = 68) of the triatomines were infected with *T. cruzi* (95% CI: 7.5–12.1%), and the infection rate was highest in the *T. brasiliensis* species complex (Table 2). The proportion of *T. cruzi-*infected triatomines was higher in the wild environment (*χ*^2^ = 134, *df* = 1, *P* < 0.001). Infected triatomines were detected in 25 municipalities, mainly in the Caatinga biome. The kernel spatial analysis showed higher density areas of *T. cruzi*-infected triatomines in the northeast and central Bahia (Fig. 3b).

**Table 2:** *Trypanosoma cruzi*-infected triatomines collected in Bahia State, Brazil, between 2013 and 2014 broken down by species

Species	*n*	+	%	*χ*^2^/Fisherʼs exact test^a^	*P-*value
*P. lutzi*	3	0	0	–	–
*P. megistus*	6	0	0	–	–
*P. tertius*	8	2	25.0	^a^	0.17
*T. brasiliensis*	57	9	15.8	2.1	0.14
*T. infestans*	80	0	0	–	–
*T. juazeirensis*	96	6	6.2	1.24	0.26
*T. lenti*	2	0	0	–	–
*T. melanica*	7	2	28.6	2.0	0.15
*T. melanocephala*	1	0	0	–	–
*T. pseudomaculata*	81	10	12.3	0.77	0.38
*T. sherlocki*	58	25	43.1	42.9	**< 0.001**
*T. sordida*	293	11	3.75	20.0	**< 0.001**
*T. tibiamaculata*	3	2	66.7	^a^	0.26

^a^Fisher’s exact test

*Note*: *P*-values < 0.05 are indicated in bold

*Abbreviations*: *n*, number of triatomines; **+**, number of *T. cruzi*-infected triatomines; %, percent of positive samples; –, data not suitable for statistical analysis

We found most bugs fed on birds (74.2%), other blood-meal sources were dogs (6%), cats (0.6%) and humans (1%) (Table 3). Triatomines that fed on birds were detected widely in Bahia State (Fig. 3), while those that fed on humans, dogs and cats were mainly detected in the northeast region, near the State of Pernambuco (Fig. 3). There was no significant difference in the frequencies of triatomines fed on cats and humans between intra and peridomestic environments (*P* > 0.05) but we detected a higher frequency of bugs that fed on dogs inside houses (*χ*^2^ = 4.07, *df* = 1, *P* = 0.04). The frequency of triatomines that fed on birds was practically the same as in the wild (86.30%) and peridomestic (86.57%) environments (*χ*^2^ = 0.013, *df* = 1, *P* = 0.9076) and statistically higher in the peridomestic environment than in the domestic environment (*χ*^2^ = 8.0, *df* = 1, *P* = 0.004).

**Table 3:** Blood-meal sources detected in triatomines collected in different environments in Bahia State, Brazil, between 2013 and 2014

Species	No. of samples			Avian			Human			Dog			Cat		
	I	P	W	I	P	W	I	P	W	I	P	W	I	P	W
*P. lutzi*	2	1	0	1	1	0	0	0	0	1	0	0	0	0	0
*P. megistus*	0	6	0	0	4	0	0	0	0	0	0	0	0	0	0
*Ps. tertius*	0	8	0	0	5	0	0	0	0	0	0	0	0	0	0
*T. brasiliensis*	33	13	11	23	11	9	3	0	0	1	1	0	0	0	0
*T. infestans*	0	80	0	0	80	0	0	0	0	0	0	0	0	0	0
*T. juazeirensis*	58	35	3	38	26	3	2	0	0	9	5	0	2	2	0
*T. lenti*	0	0	2	0	0	1	0	0	0	0	0	0	0	0	0
*T. melanica*	0	7	0	0	5	0	0	0	0	0	0	0	0	0	0
*T. melanocephala*	0	1	0	0	1	0	0	0	0	0	0	0	0	0	0
*T. pseudomaculata*	26	55	0	8	47	3	0	2	0	0	2	0	0	0	0
*T. sherlocki*	0	0	58	0	0	50	0	0	0	0	0	0	0	0	0
*T. sordida*	201	92	0	120	77	0	0	0	0	16	5	0	0	0	0
*T. tibiamaculata*	3	0	0	2	1	0	0	0	0	0	0	0	0	0	0

*Abbreviations*: I, intradomestic environment; P, peridomestic environment; W, wild environment

We detected *T. cruzi*-infected triatomines fed on humans, dogs, and cats inside houses and triatomines fed on dogs and humans in peridomestic habitats. All infected triatomines detected in the wild environment contained bird DNA.

## Discussion

The most salient findings about *T. cruzi* infection and blood-meal sources in synanthropic triatomines in Bahia were: (i) *T. cruzi*-infected triatomine bugs fed on human blood; (ii) *T. cruzi*-infected triatomines were widespread, but bugs that fed on dogs, humans, and cats were observed mainly in the northeast region; and (iii) most bugs fed on birds. These results show that triatomine bugs maintain the presence of *T. cruzi* in wild and domestic environments in the State of Bahia, Brazil.

We found 15 of the 26 recorded triatomine species in the State of Bahia during our two-year study. This result highlights the diversity of triatomines in this region [9] referring to Bahia as the Brazilian state with the highest number of triatomine species in Brazil. Ten species were recorded inside houses in sampled municipalities, mainly *Triatoma sordida*, *T. pseudomaculata,* and the *T. brasiliensis* species complex. The results differ from those observed before systematic Chagas disease vector control was carried out between 1975 and 1983 when *P. megistus* and *T. infestans* were the most captured species inside houses [5].

We observed house-invading *P. megistus* in few residences, and *T. infestans* occurred in one municipality in our study (Novo Horizonte); *T. infestans* were also recorded in other two municipalities in Bahia in the last years (Ibipeba and Tremedal) [15, 30, 31]. These results show the success in controlling domestic triatomines with the virtual elimination of *T. infestans* in Bahia municipalities [32, 33].

Our data show that *T. sordida* and *T. pseudomaculata* are the most frequently captured species in the State of Bahia, as already reported [9, 21, 34] which demonstrates their ability to colonize synanthropic environments. Other studies showed that domestic infestation with *T. pseudomaculata* increases when houses are located near preserved forests with natural ecotopes such as bird nests, tree hollows and palms [34]. Our results showed a wide distribution of *T. sordida* in the State of Bahia. A study of synanthropic triatomines in the southwest of Bahia between 2008 and 2013 [21] also showed that *T. sordida* was the most frequently captured species and presented the highest percentage of infection with *T. cruzi*. Although most specimens were captured in peridomestic habitats, as expected [9], we detected the presence of colonies and infected specimens inside houses revealing a potential risk for vectorial transmission. The other possible scenario is the risk of *T. cruzi* oral outbreaks mediated by *T. sordida* specimens inside houses, as already recorded in Bahia State [27].

Our results also revealed the high frequency of the *T. brasiliensis* species complex in Bahia. Costa et al. [35] showed high domestic infestation and infection rates for *T. brasiliensis* in Bahia between 1993 and 1999 when compared with other states of the northeast region. We detected *T. brasiliensis brasiliensis* in 20 municipalities, mainly in the northeast region of the state. To the best of our knowledge, our results show the first record of *T. brasiliensis brasiliensis* in the Bahia State. The most recent data recorded only *T. juazeirensis*, *T. melanica*, *T. lenti*, *T. bahiensis*, *T. sherlocki* and *T. petrocchiae* in Bahia [36]. Therefore, our results expand the knowledge of the geographical distribution of *T. brasiliensis brasiliensis* in northeastern Brazil. *Triatoma juazeirensis* is a recently described species [37] that was commonly misidentified as *T. brasiliensis*. Our results add new information about the behavior of this species revealing a higher number of triatomines in houses than in peridomestic habitats. We also revealed a high infection rate of *T. sherlocki* in a domestic environment in Bahia. Colonies of *T. sherlocki* were already found in houses of Bahia with *T. cruzi* infection of ~11% [38], revealing a domiciliation process and the potential risk for vectorial transmission to humans.

*Trypanosoma cruzi* has been detected in vectors in all regions of Bahia. We found high infection rates for *T. sherlocki* and *T. tibiamaculata* as already observed by Almeida et al. [38] and Ribeiro et al. [20], respectively. *Trypanosoma cruzi* infections in triatomines based on parasite morphology after optical microscopy are underestimated [39]. Consequently, the risk of *T. cruzi* transmission should be higher than the entomological-parasitological routine surveillance suggests [40, 41]. For example, test-specific naïve indices of *T. cruzi* infection in triatomines varied from 17.8%, considering only optical microscopy results, to 41.5%, considering PCR results (23.1% positive by conventional PCR and 41.3–41.4% by qPCR) [41]. Our results revealed a triatomine infection rate of approximately 10% by conventional PCR, suggesting that the triatomine infection in Bahia may be even higher than that observed in our study. These infection rates also vary according to the species and development stage of the sampled specimens. For example, *T. cruzi* infection rates observed for *T. tibiamaculata* ranged between 50–65% [20].

The infection rates of *T. cruzi* observed in our study were similar to those obtained in recent studies carried out in Pernambuco [42], Mato Grosso do Sul [43, 44] Ceará [45, 46], the Rio Grande do Norte [47, 48] and Bahia [19, 20]. Infection rates were high for *T. brasiliensis* species complex, especially for *T. sherlocki* (43.1%). Almeida et al. [47] detected *T. cruzi* in 52–71% of *T. brasiliensis* captured in Rio Grande do Norte, a higher percentage than that observed in our study in Bahia (15.8%) that could be explained by different blood-feeding habitats. The *T. sherlocki* infection rate observed in our study was four times higher than that reported by Almeida et al. [38]. Most of *T. sherlocki* specimens fed on avian blood, but the high level of infection rates of *T. cruzi* indicate an eclectic feeding behavior of *T. sherlocki*. The infection with *T. cruzi* detected in *Ps. tertius*, a species commonly associated with furnariid birds could also be explained by opportunistic feeding on mammals that eventually are found in furnariid nests. The results suggest a previous feeding of *Ps. tertius* with infected mammal blood.

*Trypanosoma cruzi* infection rates in *T. sordida* were generally less than 5% based on parasitological methods; however, Minuzzi-Souza et al. [41] estimated rates of 35% based on qPCR, a more sensitive evaluation method, which reinforces the relevance of this species as a potential *T. cruzi* vector. *Triatoma infestans* was not found to be infected by *T. cruzi*, as all the specimens were collected from a single colony into a chicken coop near the household unit; this is an unusual situation for this species, as it is considered to be exotic and domestic in Bahia.

The most frequent blood-meal source detected in triatomines in Bahia was birds (74%), similar to other studies [20, 49]. Birds are an important link in the domiciliation process of triatomines because they are common blood-meal sources in the peridomestic habitat due to be an important source of human food, through the raising of chickens, usually in the peridomicile of households [50–52]. Birds were also the main food source for *T. brasiliensis* species complex, contrasting with other studies highlighting the importance of rodents as feeding sources for *T. brasiliensis* in the Rio Grande do Norte [47, 48] and Ceará [53]. Human, dog and cat DNA, at 1%, 6%, and 0.6%, respectively, were observed less frequently. It is important to point out that previous studies have shown the key role of domestic animals in maintaining *T. cruzi* circulation within human dwellings [54, 55]. We found a higher frequency of triatomines fed on dogs inside houses highlighting the role of dogs as a potential source of *T. cruzi* in domestic transmission cycles, as already discussed [56], showing that dogs can sleep in places that are more accessible to the bugs, increasing the probability of infecting an initially uninfected bug.

The species found with human DNA were *T. brasiliensis*, *T. juazeirensis,* and *T. pseudomaculata*, and several other studies have revealed that these species are capable of transmitting *T. cruzi* to humans in the domestic environment [34, 35, 37, 48, 53, 57]. Regarding the spatial distribution of *T. cruzi* and blood-meal sources, we observed clusters of infected triatomines that fed on humans and domestic animals in the municipalities of Curaçá and Irecê, located in the northeast region of Bahia. Simulations of vulnerability to *T. cruzi* vector-borne transmission in Brazil based on the most prevalent species also have indicated the northeast region of Bahia as having high vulnerability to the vector-borne transmission of *T. cruzi*; vulnerable municipalities exhibited a higher occurrence of synanthropic triatomines, lower socioeconomic levels (high percentage of properties in rural areas with individuals living in extreme poverty), and more extensive anthropized areas (percentage of deforested area in the municipality) [10].

## Conclusions

Triatomines remain widely distributed in Bahia, with relevant *T. cruzi* infections and feeding on human and domestic animals inside houses, mainly in the northeast region of Bahia, thus maintaining the potential risk of *T. cruzi* transmission to humans. Ten species were recorded inside houses, mainly *Triatoma sordida*, *T. pseudomaculata,* and the *T. brasiliensis* species complex. Molecular and spatial analysis are useful to reveal *T. cruzi* infection and blood-meal sources in synanthropic triatomines, identifying areas with an ongoing threat for parasite transmission and improving entomological surveillance strategies.

## Supplementary information

**Additional file 1: Figure S1.** Notified and confirmed acute cases of Chagas disease in Brazil. **Table S1.** Confirmed cases of acute Chagas disease in Brazil between 2016 and 2019.** Table S2.** Reagents used for the amplification of molecular targets.** Table S3.** Thermocycling conditions used for amplification of molecular targets.** Table S4.** Sequences of the primers used.

## Data Availability

Data supporting the conclusions of this article are included within the article and its additional file. The datasets generated and/or analyzed during the present study are available from the corresponding author on reasonable request.

## Abbreviations

PCR: polymerase chain reaction
SESAB: Epidemiologic Surveillance team of Bahia State Health Service
DNA: deoxyribonucleic acid
ITS2: internal transcribed spacer 2
GPS: Global Position System
GIS: Geographical Information System
qPCR: quantitative polymerase chain reaction
SINAN: Notification Disease Information System
