# Supplementary material for: Wide distribution of Trypanosoma cruzi-infected triatomines in the State of Bahia, Brazil
Source: Parasit Vectors. 2019 Dec 26;12:604. doi: 10.1186/s13071-019-3849-1 (PMC6933904; doi:10.1186/s13071-019-3849-1)
Supplement: Supplementary file 1 — Additional file 1: Figure S1. Notified and confirmed acute cases of Chagas disease in Brazil. Table S1. Confirmed cases of acute Chagas disease in Brazil between 2016 and 2019. Table S2. Reagents used for the amplification of molecular targets. Table S3. Thermocycling conditions used for amplification of molecular targets. Table S4. Sequences of the primers used. [file 13071_2019_3849_MOESM1_ESM.docx]

**Additional file 1**

**Epidemiological information of Chagas disease in Brazil**

**Figure S1. Notified and confirmed acute cases of Chagas disease in Brazil between 2007 and 2016.**

**
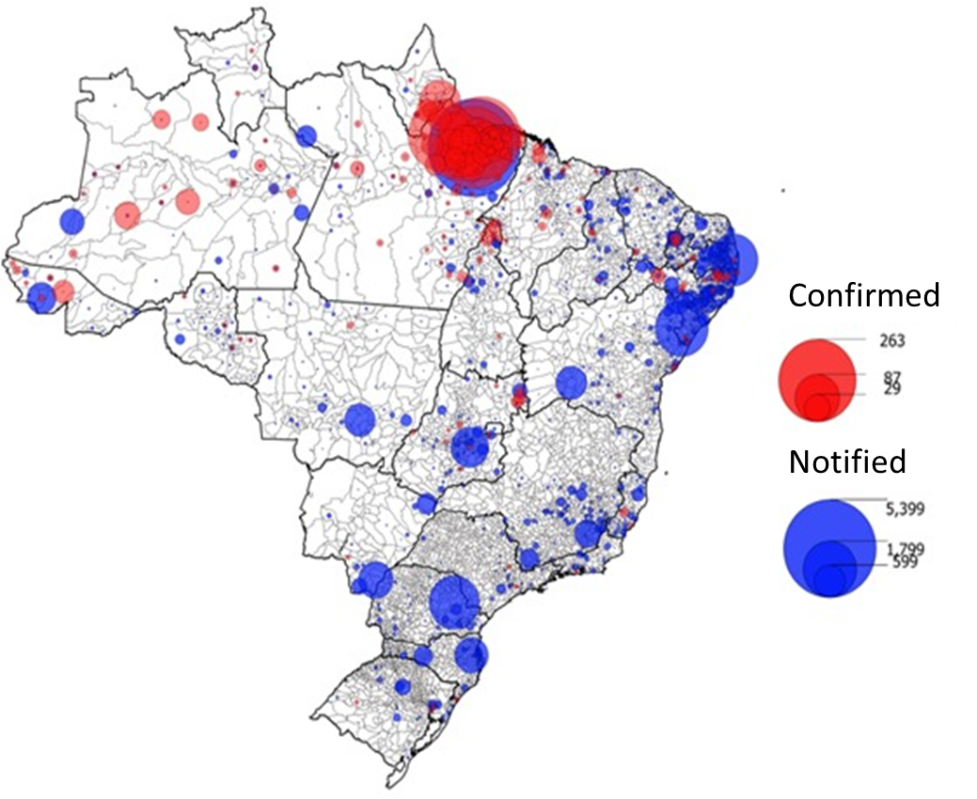
**

**Source**: SINAN - Notification Disease Information System of Brazil.

**Table S1. Confirmed cases of acute Chagas disease in Brazil between 2016 and 2019.**

**
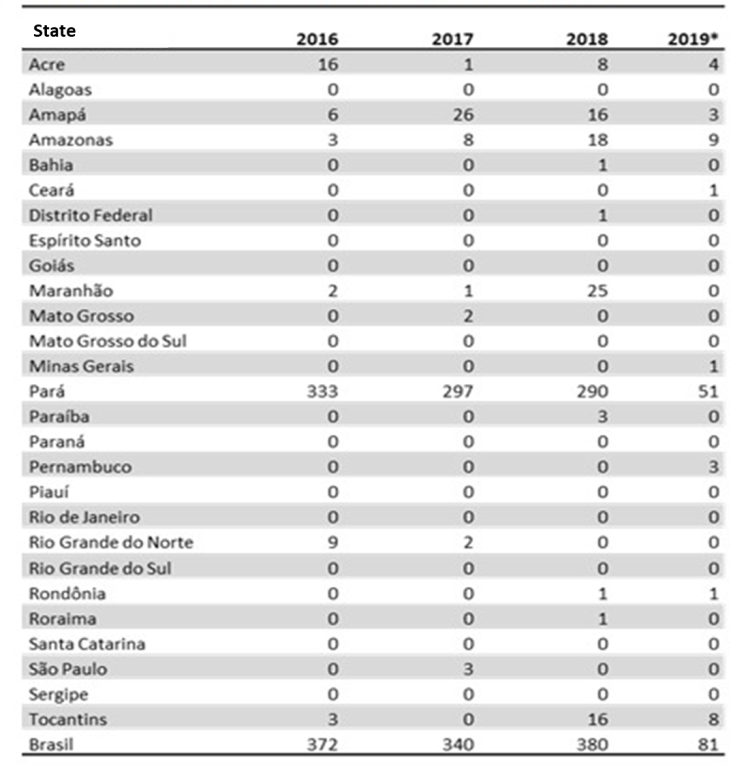
**

**Source**: SINAN - Notification Disease Information System of Brazil.*until 28º epidemiologic week.

**Reagents and conditions for PCRs**

**Table S2. Reagents used for the amplification of molecular targets**

| **Reagents** | **Final concentration** | **Tube** |
| --- | --- | --- |
| **QIAGEN MASTER MIX** | **1x** | **12.5** |
| **PRIMER A** | **0.1 uM** | **0.5** |
| **PRIMER B** | **0.1 uM** | **0.5** |
| **Electrophoresis dye** | **1x** | **0** |
| **H2O** | **-** | **10.5** |
| **DNA (ng/uL)** | **~100ng/uL** | **1** |
| **Total volume (uL)** | **-** | **25** |

**Table S3. Thermocycling conditions used for amplification of molecular targets**

| **Thermocycler** | **Temp (°C)** | **Time (seg.)** |
| --- | --- | --- |
| **Temperature of lid** | **94** | **-** |
| **T1 - Denaturation 1** | **94** | **180** |
| **T2 - Denaturation 2** | **94** | **30*** |
| **T3 – Annealing** | **60** | **30*** |
| **T4 – Extension** | **72** | **60*** |
| **T5 – Final Extension** | **72** | **600** |
| **At the end keep at** | **12** | ∞ |

Legend: *40 cycles.

**Table S4. Sequences of the primers used**

| **Target** | **Name** | **Specificity** | **Sequence** | **Nucleotides** | **Amplicon** |
| --- | --- | --- | --- | --- | --- |
| **Amplification**  **Control** | **ITS2-F** | **Triatominae** | **5’- CTAAGCGGTGGATCACTCGG -3’** | **20** | **127** |
|  | **ITS2-R** | **Triatominae** | **5’- GCACTATCAAGCAACACGACTC -3’** | **22** |  |
| ***T. cruzi*** | **COII-F** | ***T. cruzi*** | **5’- CCA TAT ATT GTT GCA TTA TT -3’** | **20** | **375** |
|  | **COII-R** | ***T. cruzi*** | **5’- TTG TAA TAG GAG TCA TGT TT -3’** | **20** |  |
|  | **Miniexon-F** | ***T. cruzi*** | **5´- AAG GTG CGT CGA CAG TGT GG -3´** | **20** | **150-200** |
|  | **Miniexon-R** | ***T. cruzi*** | **5´- TTT TCA GAA TGG CCG AAC AGT -3´** | **21** |  |
|  | **Rdna-F** | ***T. cruzi*** | **5´- CTC CCC AGT GTG GCC TGG G -3'** | **19** | **110,119, 125** |
|  | **Rdna-R** | ***T. cruzi*** | **5´- CGT ACC AAT ATA GTA CAG AAA CTG -3'** | **24** |  |
| **Blood meal** | **Human-F** | ***Homo sapiens*** | **5' - GTA GTA CAT AAA AAC CCA ATC CAC ATC - 3'** | **27** | **470** |
|  | **Human-R** | ***Homo sapiens*** | **5' - GTC GGA TAC AGT TCA CTT TAG CTA CC - 3'** | **26** |  |
|  | **Dog-F** | ***Canis familiaris*** | **5' - GTC AAT GGT TTC AGG ACA TAT AGT TTT - 3'** | **27** | **476** |
|  | **Dog-R** | ***Canis familiaris*** | **5' - TAT TGT ATG CAC TTA GTC CTG TTT TTG - 3'** | **27** |  |
|  | **Cat-F** | ***Felis catus*** | **5' - ACA GGA TCA GAA ACC TTT ATC TGA CTA - 3'** | **27** | **1179** |
|  | **Cat-R** | ***Felis catus*** | **5'- TTT GAC TTA AAA TTT ATG GTT TGG TTT - 3'** | **27** |  |
|  | **Avian-F** | **Class Avian** | **5' - ATA GAA TGG CCT GGG TTG AAA AG - 3'** | **23** | **197** |
|  | **Avian-R** | **Class Avian** | **5' - AAG TTT TTC ACA CAG AGG GTG GT - 3'** | **23** |  |

***Abbreviations***: *T. cruzi*, *Trypanosoma cruzi*; *T. brasiliensis*, *Triatoma brasiliensis*; PCR, Polymerase Chain Reaction; *T. infestans*, *Triatoma infestans*; SESAB, Epidemiologic Surveillance team of Bahia State Health Service; DNA, Deoxyribonucleic acid; ITS-2, Internal transcribed spacer - 2; GPS, Global Position System; GIS, Geographic Information System; *T. sordida*, *Triatoma sordida*; *T. pseudomaculata*, *Triatoma pseudomaculata*; *P. megistus*, *Panstrongylus megistus*; *T. juazeirensis*, *Triatoma juazeirensis*; *T. sherlocki*, *Triatoma sherlocki*; qPCR, quantitative Polymerase Chain Reaction; *T. tibiamaculata*, *Triatoma tibiamaculata*;
